# Scaling up newborn care technologies from tertiary- to secondary-level hospitals in Malawi: an implementation case study of health professional perspectives on bubble CPAP

**DOI:** 10.1186/s43058-020-00092-8

**Published:** 2020-11-04

**Authors:** Mai-Lei Woo Kinshella, Sangwani Salimu, Tamanda Hiwa, Mwai Banda, Marianne Vidler, Laura Newberry, Queen Dube, Elizabeth M. Molyneux, David M. Goldfarb, Kondwani Kawaza, Alinane Linda Nyondo-Mipando

**Affiliations:** 1 Department of Obstetrics and Gynaecology, BC Children’s and Women’s Hospital and University of British Columbia, Vancouver, Canada; 2 Department of Pediatrics and Child Health, College of Medicine, University of Malawi, Blantyre, Malawi; 3 Queen Elizabeth Central Hospital, Pediatrics, Blantyre, Malawi; 4 Department of Pathology and Laboratory Medicine, BC Children’s and Women’s Hospital and University of British Columbia, Vancouver, Canada; 5 School of Public Health and Family Medicine, Department of Health Systems and Policy, College of Medicine, University of Malawi, Blantyre, Malawi

**Keywords:** Malawi, Newborn care, Implementation, Tertiary- and secondary-level care differences, Bubble continuous positive airway pressure (CPAP), Health worker perspectives

## Abstract

**Background:**

While Malawi has achieved success in reducing overall under-five mortality, reduction of neonatal mortality remains a persistent challenge. There has, therefore, been a push to strengthen the capacity for quality newborn care at district hospitals through the implementation of innovative neonatal technologies such as bubble continuous positive airway pressure (CPAP). This study investigates tertiary- versus secondary-level hospital differences in capacities for bubble CPAP use and implications for implementation policies.

**Methods:**

A secondary analysis of interviews was conducted with 46 health workers at one tertiary hospital and three secondary hospitals in rural Southern Malawi. Grounded theory was utilized to explore the emerging themes according to health worker cadres (nurse, clinician, district health management) and facility level (tertiary- and secondary-level facilities), which were managed using NVivo 12 (QSR International, Melbourne, Australia).

**Results:**

We identified frequent CPAP use and the availability of neonatal nurses, physicians, and reliable electricity as facilitators for CPAP use at the tertiary hospital. Barriers at the tertiary hospital included initiation eligibility disagreements between clinicians and nurses and insufficient availability of the CPAP machines. At secondary-level hospitals, the use was supported by decision-making and initiation by nurses, involving caretakers to assist in monitoring and reliable availability of CPAP machines. Bubble CPAP was hindered by unreliable electricity, staffing shortages and rotation policies, and poor systems of accountability.

**Conclusion:**

While this study looked at the implementation of bubble CPAP in Malawi, the findings may be applicable for scaling up other novel neonatal technologies in low-resource settings. Implementation policies must consider staffing and management structures at different health services levels for effective scale-up.

Contributions to the literature

- Effective implementation of neonatal technologies to strengthen newborn care in low-resource settings is key to improving quality of care and reducing newborn deaths. While some studies explore the implementation process at tertiary-level facilities, there remains a gap in understanding how barriers and facilitators to implementation may differ in rural, secondary-level health facilities.
- Although we found a number of similarities in barriers and facilitators to implementation between urban tertiary-level facilities and rural secondary-level facilities around staffing, training, and infrastructure, our findings revealed different staffing and management structures at the different health service levels that must be considered for effective implementation.
- These findings contribute to the literature by presenting implementation factors involved in the introduction of technologies from a tertiary-level hospital in a low-resource health system to secondary-level hospitals where resources may be even more constrained. Our findings highlight the importance of workplace social norms and strengthening self-efficacy to promote an enabling environment for the uptake of neonatal care technologies.

## Background

Malawi has achieved a number of successes in maternal and child health including meeting the Millennium Development Goal of reducing under-five child mortality rates [1] and an increase in the proportion of facility births from 55% in 2010 to 91% in 2016 [2]. However, the high burden of neonatal deaths continues to be a challenge [1, 3]. Increases in facility births were not accompanied by reductions in neonatal mortality, which has remained at a rate of 27 per 1000 live births between 2004 and 2016 [2, 4]. A Malawian study found that health facilities with poor quality obstetric and newborn care were concentrated in rural areas that were associated with 2.3% higher neonatal mortality rates [5]. To reduce the unacceptable burden of newborn deaths in Malawi, there has been a push to improve the quality of newborn care at district levels.

One in every ten infants are born premature in Malawi (10.5%), and while preterm survival rates have increased in high-income countries, preterm newborns still die in resource-limited health settings due to a lack of adequate newborn care [6]. The World Health Organization (WHO) strongly recommends continuous positive airway pressure (CPAP) to manage respiratory distress syndrome (RDS) among preterm infants [7], who are extremely vulnerable due to immature respiratory system [8, 9]. A systematic review found that bubble CPAP is a safe way of delivering CPAP in low- and middle-income countries (LMICs) and was effective in reducing the need for mechanical ventilation [10]. Though there is evidence for the safety and efficacy for bubble CPAP for neonatal care in LMICs, a gap exists in implementation. A systematic review of barriers and facilitators to bubble CPAP implementation in sub-Saharan Africa highlighted concerns about the reliable availability of equipment, difficulties in engaging and informing caregivers, and staffing shortages, particularly in rural, secondary-level hospitals [11]. The review found that most studies were conducted in tertiary-level facilities with few at secondary-level facilities. The few conducted at secondary-level facilities reported use of decision aids as clinicians were very few in number [11]. Implementation factors and constraints in district hospitals may compromise the benefits of bubble CPAP.

The healthcare system in Malawi is divided into 28 districts, and each district is managed by a district health management team (DHMT). The DHMT includes the district health officer (DHO), district medical officer (DMO), and district nursing officer (DNO), who oversee the overall health management and clinical and nursing activities in the district [12]. Only the DMO is required to be a physician, and consequently, there may be only one physician in the whole district. While overseen by the DHMT, district hospital wards are led by nurses and non-physician clinicians (clinical officers) [13]. In addition to government district hospitals and mission hospitals run by the Christian Health Association of Malawi (CHAM) that provide secondary-level care in rural areas, there are four central hospitals providing tertiary-level care [12]. Staffing for neonatal care at a central hospital includes consultants (pediatricians), registrars (pediatricians in training), interns (doctors in training), clinical officers, nurses, and lay patient attendants.

Understanding the health system in Malawi is important for considering the scale-up of a low-cost, stand-alone bubble CPAP system named Pumani developed by Rice University (Houston) in partnership with the University of Malawi and initially trialed at Queen Elizabeth Central Hospital in Blantyre, Malawi [14]. The Pumani is 15 times less costly than other commercial CPAP machines [14], and the use of the Pumani was scaled up across the country from 2012 to 2017 in all government hospitals, as well as eight mission hospitals. As nursery facilities are strengthened in district-level hospitals, there is a need to understand how implementation factors for more advanced treatment approaches may vary between an urban tertiary-level care setting and a rural secondary-level care setting. This research was a part of a larger project, “Integrating a Neonatal Healthcare Package for Malawi” (IMCHA #108030), which seeks to understand how the scale-up of low cost and locally appropriate innovations to improve newborn care at low-resource health facilities can be effectively implemented. The purpose of this qualitative study was to investigate the key areas where tertiary and district hospitals differed in their health system and service delivery and discuss the implications for implementation of bubble CPAP and other novel neonatal technologies.

## Methods

### Study design

The project is part of the Innovating for Maternal and Child Health in Africa (IMCHA) initiative funded by the Canadian International Development Research Centre (IDRC), the Global Affairs Canada (GAC), and the Canadian Institutes for Health Research (CIHR). This is a secondary analysis of data from a qualitative study using in-depth interviews with healthcare workers regarding bubble CPAP. The interviews explored experiences of training, initiation, monitoring, and perspectives of health workers and caregivers at Malawian hospitals. The study is reported based on the “Consolidated Criteria for Reporting Qualitative Research” (see Additional file 1 for the checklist) [15].

### Research context

The research was conducted in Southern Malawi at three secondary-level hospitals that served as rural referral centers for their districts, each with populations between 400,000 and almost 700,000 as of the 2018 census, and one tertiary hospital, which served the southern region of Malawi together with another central hospital [16]. Two of the three district hospitals are government facilities (district hospitals 1 and 3) while one hospital (district hospital 2) is run by CHAM. All hospitals provide essential maternal and child health services free of charge [13]. All hospitals in this study had labor and delivery, cesarean and nursery facilities, and admitted small and sick newborns [17]. None of the district hospitals was staffed by a full-time obstetrician-gynecologist or pediatrician, and cesarean deliveries were performed by clinical officers or general physicians [17]. The pediatric department of the tertiary hospital has a neonatal care unit as well as several high-dependency units (HDU) staffed by pediatricians, registrars, interns, registered nurse midwives, and nurse midwife technicians [13]. Bubble CPAP is administered in the nursery unit and pediatric nursery ward in the tertiary hospital and in small rooms within nursery units at the district hospitals [13, 18]. For more information on newborn health services and quality of care, see the facility assessment of the district facilities (Kawaza et al. [13]).

### Data collection

Thirty to 60-min in-depth interviews were conducted with 46 nurses, clinicians, and district health management between June and August 2018. Hospitals and healthcare workers were purposefully sampled to obtain a range of experiences and views. Facilities were selected in consultation with the Malawian Ministry of Health to represent different health management structures and different geographical health services zones. Health professionals involved in the care and/or decision-making for newborns at the four health facilities were recruited to share a diversity of perspectives related to the implementation of bubble CPAP. Based on the numbers of healthcare professionals that engaged with bubble CPAP for neonatal care reported in a facility assessments study [17], a sample size of 10–15 participants at each site was estimated to achieve data saturation [13]. A team of Malawian research assistants conducted interviews using a pre-tested, semi-structured guide in English or the local language of Chichewa depending on the participant’s preference (see Additional file 2 for the topic guide). A detailed description of the qualitative research process is described elsewhere (see Nyondo-Mipando et al. [13]).

### Data analysis

Interviews were audio-recorded with permission, transcribed verbatim, and translated into English from Chichewa where necessary. Qualitative analysis was undertaken by a local Malawian research assistant (SS) and a Canadian qualitative researcher with a background in medical anthropology (MWK) and overseen by a Malawian qualitative research and health systems expert (ALNM). Grounded theory was utilized to inductively explore the emerging themes and develop a coding framework for analysis using the NVivo 12 software (QSR International, Melbourne, Australia) [19–21]. Grounded theory was used to highlight the lived experiences and issues significant from the perspective of the participants interviewed. It features an iterative process of analysis and interpretation that builds from the initial coding labeling excerpts from the transcripts to focused coding on issues that appear most frequently and/or are the most relevant to the research question [19–21]. Further analysis by health service level emerged as a topic of investigation in the discussion of primary research findings with clinicians at pediatric departmental meetings at Queen Elizabeth Central Hospital, Blantyre, Malawi. Primary coding of the entire dataset was completed by one research (SS), which was reviewed by two researchers (MWK and ALNM) to verify for soundness and completeness, and a secondary analysis was undertaken by MWK to re-analyze by health worker cadres (nurse, clinician, district health management) and facility level (secondary- and tertiary-level) and reviewed by ALNM for reliability.

## Results

Of the 46 interviews conducted, there were 16 from the tertiary hospital and 10 each from the three district hospitals. Nine DHMT personnel (two DHO, four DMO, three DNO) along with four clinical officers and 16 nurses from district hospitals were interviewed. From the tertiary hospital, 13 nurses and three pediatric specialists (consultant and registrars) were interviewed. The health workers interviewed reported a median of 3 years of experience using bubble CPAP [IQR 1.2, 4] and 8 years as the median length of service as a health worker. For a detailed description, see Nyondo-Mipando et al. [13].

### Training

#### Training models

Both tertiary- and secondary-level hospitals used a train-the-trainer model. The tertiary hospital nursery was a key site of training for nurses from the district hospital. District hospital nurses described the 5-day mentorship training at the tertiary hospital nursery as very useful. However, some nurses at the tertiary hospital expressed the need to update their own knowledge as their knowledge gaps were passed on to the district nurses who trained with them.

I went to Queens, Chatinkha Nursery where we were taught about bubble CPAP by peers. We were there for 5 days… When I went there, I had no knowledge about CPAP so when I went there I was taught a lot so that when I was coming I was not the same as when I was going. District 3 hospital nurse, F, 26

There are a lot of people coming every day to work at this hospital, so they also need to be trained instead of being trained by us…..[I]f that person has been taught by someone who is not competent enough to put the baby on CPAP then it becomes a problem because he/she will be doing things in a wrong way…. The old ones will also need it (refresher training) so that they should be able to have or gain the knowledge of new things or new developments about CPAP. Tertiary hospital nurse, F, 50

#### Frequency of practice

Frequent use of CPAP at the tertiary hospital strengthened training. Eleven of 16 (69%) respondents at the tertiary hospital said they used bubble CPAP daily (see Table 1). In contrast, none of the respondents from the three district hospital sites reported using bubble CPAP daily, and 13 of 30 (44%) said they never used it or used it less than twice a month.

**Table 1:** Responses by health workers on the frequency of using bubble CPAP in their facilities

Site	Less than once a month or never	Once or twice a month	Once or twice a week	Three to six times a week	Daily	Other responses^a^
District hospital (*n* = 30)	8/30 (27%)	5/30 (17%)	11/30 (37%)	2/30 (7%)	0/30 (0%)	4/30 (13%)
Tertiary hospital (*n* = 16)	0/16 (0%)	0/16 (0%)	2/16 (13%)	3/16 (19%)	11/16 (69%)	0/16 (0%)

^a^Other responses included “often” (one response from district hospital 1), “rarely” (one response each from district hospitals 1 and 2), “whenever the baby needed CPAP” (one response from district hospital 3). These responses could not be clearly quantified

Frequent hands-on practice, as well as witnessing its benefits, motivated nurses to continue using bubble CPAP. In contrast, infrequent use of bubble CPAP led to further infrequent use at district hospitals.

I would say we learned most of the things here at the ward because we are practicing it, we are doing it and we could see how it is benefiting the babies, so that alone motivated us. Central hospital nurse, F, 29

No, I have never done it (put a baby on CPAP)…the problem is that we also rarely receive babies in need of CPAP. Right now I cannot manage to commence a baby on CPAP on my own. District hospital 1 nurse, F, 38

I think there are days where we literally have no baby needing CPAP…I think we can even go a week without a baby initiated on CPAP… Because here we are not frequently getting babies needing CPAP…so people will sort of lose the skill. District hospital 1 DMO, M, 27

### Initiation

#### Deciding to use bubble CPAP

There were differences between tertiary- and secondary-level hospital staff in decision-making to initiate bubble CPAP. The tertiary hospital featured a more rigid hierarchy with clinicians considered as the decision-makers, supported by the reliable availability of doctors in the ward. In critical situations, trained nurses may initiate bubble CPAP but would follow-up with a clinician. Adequate training to understand the entire medical condition and the ability to order diagnostic tests, as well as workflow, were described as reasons why clinicians were considered as decision-makers by nurses at the tertiary hospital.

The doctors are always available in the wards and when we see that the condition is not good, we always consult the doctors and they are able to order the CPAP in the process. Tertiary hospital nurse, F, 38

I think it’s also the order of authority and most of the times…we don’t prescribe anything. We only carry out some of the things that the doctors prescribe… I think if people were just prescribing these, maybe it would cause a lot of problems, so everybody has their place and they know what to do…. [M]aybe a nurse may discover some of the gaps but they would consult the doctor…so it’s about order, just order, to make things work things in an orderly manner. Tertiary hospital nurse, F, 35

At district hospitals, clinicians were considered as decision-makers, but because there were so few clinicians, shortfalls in staff led to nurses empowered to initiate CPAP. Clinical officers were often busy in multiple wards or in the operating theater. Skill and knowledge on CPAP eligibility and initiation were prioritized over authority structures.

When the clinician is around, he authorizes to put the baby on CPAP but sometimes when he is not around, we make the decision of putting the baby on CPAP… I think mostly, we decide… [W]e have two clinical officers…allocated in post-natal and labour ward …[but] sometimes he is in theatre…sometimes he is busy in the labour ward. So when we know that we can assist this baby, we do it. District 1 nurse, F, 30

We have fewer clinicians. Usually clinicians will have to go through several wards… usually the clinician will assess them in the morning and afternoon or just in the morning…[It] is the nurse who is constantly in the wards, who monitors these babies… I don’t think is an issue of authority. It’s just an issue of who knows how to initiate and to say which baby would benefit from CPAP. District hospital 1 DMO, M, 27

#### Disagreements about initiation

Disagreements between nurses and clinicians around CPAP initiation were more frequently described at the tertiary hospital, particularly around the eligibility of premature newborns with birth asphyxia. While clinicians were decision-makers, nurses were often the ones who were trained to use algorithms such as TRY (Tone: good; Respiratory distress; Yes, heart rate above 100 beats/min). Nurses described how a newborn with a low Apgar score should not be put on CPAP, but clinicians argued that low scores could be due to lung underdevelopment in premature infants and could consequently benefit from CPAP. Sometimes when nurses felt strongly about a contraindication, they sought a second opinion from another clinician. In some cases, nurses would leave the equipment for clinicians to avoid initiating CPAP.

A child with severe HIE (Hypoxic Ischemic Encephalopathy) is not active; he will be floppy…The doctors would still insist that we put the baby on CPAP…while we learnt that…the problem in the brain…. Most of the times, when we document that, “I will not commence this one on CPAP”, there will be conflicts… I do not know if the doctors either do not know this, or they are not told, or they just feel that whatever they say, nurses should just follow. Tertiary hospital nurse, F, 29

With birth asphyxia, sometimes they (doctors) say, “The baby is working very hard. Please just commence CPAP on that baby” yet the Apgar score was very low, 2/10, 3/10, so nurses refuse to put baby on the CPAP. They just assemble the equipment until the doctor does himself. Tertiary hospital nurse, F, 49

Sometimes when the babies are coming from the labor ward, they are given low Apgar score…. So most of the times when people see a low Apgar score, they conclude… that this is birth asphyxia even when that baby is a premature…a premature baby cannot have birth asphyxia, so sometimes, its misdiagnosis …can lead to a failure to put that baby on CPAP. Tertiary hospital pediatrician, M, 42

While there were some disagreements at district hospitals, it was not as frequent as described at the tertiary hospital. Clinical officers tended to defer to the decision of nurses as they had more experience and had received more CPAP training.

The clinician sometimes says that we can put the child [but] the nurse says, no, we cannot put this child [on CPAP] because he has really, really low Apgar score…. The clinician was not trained but the nurse was trained. District hospital 1 nurse, F, 31

[S]ometimes, you really feel this baby can really benefit from CPAP but then criteria is not allowing him…So when you prescribe CPAP, nurses do not agree, so you decide that if that is the case, then I am not taking that responsibility. District hospital 2 clinical officer, M, 36

#### Accountability and follow-up

In addition to staffing and decision-making differences between tertiary- and secondary-level district hospitals, initiation was also influenced by different levels of accountability and follow-up. Nurses at the tertiary hospital shared that junior clinicians complained to senior clinicians if nurses delayed initiation. At the district hospitals, clinical officers did not always follow up to see if their order of CPAP was actually completed.

When he (doctor) was ordering, he thought there were machines available while in fact the machines were not functioning. He even reported to the head doctor that he ordered CPAP, but the nurses did not initiate it. Tertiary hospital nurse, F, 29

This ward is busy. We have labour ward postnatal, nursery and antenatal so… we do ward rounds fast and just order …We may not even come back after ward rounds because we are busy… Most times, we just prescribe and move on… Whether they put [baby on CPAP] or not… follow up is not easy …. I don’t remember the final result; it was a weekend and I was away. District 3 clinical officer, M, 36

### Monitoring

#### Coping with staffing shortages

Nurses acknowledged that CPAP required frequent monitoring and that monitoring was ideally completed after the first 15 min, 1 h after initiation, and then every 4 to 6 h. However, nurses in both health settings described shortfalls in staff coverage as challenges to monitoring. In response to staffing shortages at the tertiary hospital, senior nurses would allocate nurses to different responsibilities to ensure adequate monitoring. The availability of clinicians also supported monitoring as they reviewed the newborns’ condition during their rounds.

The standard ratio of nurse to patient is supposed to be one-to-one for a patient to be well looked after …but here because of shortage, you would find that on duty we will be three nurses against sixty patients…So, it just requires you to prioritize…Someone would [be] on the orders, someone on the admissions. We are supposed to divide our time otherwise there will be more deaths…The areas that children are on CPAP…they are the sick babies. Your priority should be towards the sick babies, whether the babies are ten or more, you can divide your time… Tertiary hospital nurse, F, 38

There were fewer admissions to secondary-level hospitals, but district health workers discussed how staffing shortages resulted in nurses being reallocated to the labor and post-natal wards. Staffing shortages and burdens of CPAP monitoring were discussed alongside a hesitancy to initiate CPAP.

CPAP is involving, unlike just putting neonates on mere oxygen therapy. For us to have a good result on bubble CPAP, we need monitoring… like half-intensive care… We don’t put a lot of neonates on bubble CPAP because of workload for CPAP really requires monitoring… Since we only have one nurse during the night, we will not have proper monitoring and as a result, might die while on bubble CPAP so let’s just leave them on mere oxygen therapy. District hospital 3 nurse, M, 30

It (monitoring) is supposed to be one hour then every four hours but that does not happen because of shortage of staff. This is killing us… Like today, we have two (nurses) who are supposed to check the postnatal, babies, nursery, women who had caesarean…Sometimes the whole maternity may depend on one clinician. District hospital 1 clinical officer, M, 31

#### Caregivers as a resource

At the districts, caregivers were seen as a resource for monitoring and caring for their baby while on CPAP. This was not possible at the central hospital where the nursery had restricted visiting hours.

We also advise the mother in order for her to take part in observing. It’s not like delegating the duty to her but just to be careful in observing the things happening whenever the nurses are absent from the ward. District hospital 2 nurse, F, 38

If we explain to the mothers well and if they observe that things are not working as they were told, mothers can alert the nurses to say this thing is not the way you told us it will work. It helps most of the times. District hospital 3 DMO, F, 39

### Hospital setting

#### Infrastructure and equipment

Health workers described challenges of other equipment, such as oxygen supply and supplies requiring appropriate sizing (such as nasal prongs). Medical staff at the tertiary hospital reported the challenge of not having enough CPAP machines, while the lack of reliable electricity was a key challenge at district hospitals. District hospital staff reported daily electricity outages, often many hours in length. Even with a generator, there were delays and issues around the lack of fuel.

We have few CPAP machines in this ward and yet you would find that there are 4 children who are supposed to be on CPAP that day or here comes the 5th one to be put on CPAP as well and yet the machines are not enough and you would go to the other wards to look for the spare ones…but then you wouldn’t find any spare machine there, and you will see that all the machines are in use hence the delay. Tertiary hospital nurse F, 38

Whenever you put CPAP, you make sure that electricity is there… It is almost daily that we have blackouts… [For the] generator to be started, it requires…to be authorised by management. So it’s quite challenging you find that the baby needs CPAP and there in no power… we go to director to negotiate… District hospital 2 clinical officer, M, 36

## Discussion

Interviews with health workers at three secondary-level hospitals and one tertiary hospital in Southern Malawi highlighted that district hospital nurseries are not simply smaller versions of their tertiary hospital counterparts. Staffing and management structures are different, which presents unique barriers and facilitators (see Table 2).

**Table 2:** Summary of barriers and facilitators by hospital level

	Barriers	Facilitators
**Tertiary hospital**	• Disagreements between clinicians and nurses (authority and training) • Insufficient availability of CPAP machines	• Frequent use of strengthened training and experience • Nurses dedicated to nursery • Reliable availability of physicians • Reliable availability of electricity
**District hospitals**	• Lack of reliable electricity • Training gaps exacerbated by staff rotation • Severe staffing shortages • Unsupportive environment to strengthen self-efficacy • Poor systems of accountability	• Empowering nurses as decision-makers and initiators • Involving caretakers to help monitor • Availability of CPAP machines

Factors that helped facilitate bubble CPAP use at the tertiary hospital included frequent use, nurses dedicated to the nursery, and the reliable availability of physicians and electricity. Frequent use demonstrated the efficacy of the system to save lives, which supported nurses’ motivation to use CPAP and strengthened an institutional culture of using CPAP. Physician follow-up on their CPAP orders promoted accountability. Poor motivation and accountability were described as key barriers in bubble CPAP use in a previous implementation study in Ethiopia [22] while strong leadership in neonatal care by health facility management was an important facilitator of effective bubble CPAP use in a study of 19 hospitals in Kenya [23]. At the tertiary hospital, there is more access to specialists with the capacity for individualized decision-making around care; however, this may be a source of friction and display of power between cadres of health workers. Formal bubble CPAP training included decision-making algorithms and recruited senior nurses as direct users of the system and mentors for junior nurses from district hospitals. Their experiences and training sometimes conflicted with the conceptualization of clinicians as the decision-makers and authority on patient management. The example of nurses assembling the supplies but leaving the physician to take the final steps in initiating CPAP to demonstrate their protest vividly describes power relations and the key issue of responsibility at the tertiary facility.

Factors that helped facilitate bubble CPAP utilization at district hospitals included empowering nurses to initiate CPAP to cover for clinician shortages, involving caretakers to help monitor and reliable availability of machines. Health workers at district hospitals emphasized barriers of training gaps exacerbated by staff rotations and shortages. Studies of bubble CPAP in Malawi [24], Uganda [25], and Kenya [26] also found that nursing shortages in rural district hospital nurseries were a key barrier and raised the concern that bubble CPAP could exacerbate demands on health workers. Interviews with staff at district hospitals highlighted the requirement of nurses’ internal motivation to prioritize bubble CPAP use within their time and labor capacities and accept the responsibility for more frequent monitoring and elements of personal confidence and innovativeness to try novel systems. Consequently, though nurses were more empowered to initiate CPAP at district-level hospitals, this may not be feasible without an enabling environment including adequate training, support from clinicians, and adequate staffing in the nursery. Without these, there may be lower initiation rates of eligible infants, as reported by a Rwandan study in rural district hospitals with high staff turnover, no full-time specialist, and gaps in respiratory distress identification where only around half of the eligible preterm newborns were provided CPAP (49 of 92 eligible infants) [27]. Heavy demands on clinicians meant that follow-up on bubble CPAP utilization and accountability were important challenges.

### Implications for policy and practice

Instituting similar bubble CPAP policies at tertiary- and secondary-level health facilities may result in misaligned implementation approaches due to different staffing and management structures. For tertiary hospital settings, where there are specialized health professionals and potential fiction between cadres, a potential strategy to strengthen common understandings of CPAP is to train nurses and physicians together. Additionally, workplace cultures valuing teamwork could be modeled by senior clinicians and hospital leadership to mitigate power dynamics where inexperienced trainee doctors may feel threatened by experienced nurses. Though important across all health settings, strengthening knowledge and skills around weaning the infant off bubble CPAP was especially an issue in the tertiary hospital where there was a high demand for the devices, and participants frequently reported shortages.

For district hospital settings, in particular, study results suggest moving away from a train-the-trainer model. There were frequent reports that trained nurses were rotated to other wards, and those left in the nursery to train others may not be confident or knowledgeable. Training videos instead may support consistent delivery of information, accompanied by case examples and hands-on practice using models, as cases may not be frequent enough for the institutionalization of the practice. Empowering nurses to initiate bubble CPAP, especially in nurse-led wards in district hospital settings, requires formal, written policy that outlines roles and responsibilities and an enabling environment. This includes the development of checklist guides for the initiation and monitoring to support both the process of making a decision as well as justification for decisions made when reviewed by senior staff and accountability of practices. Hospital management leadership and commitment to the prioritization of quality newborn care and supportive supervision are also needed. Continued support could be delivered through mobile or online platforms, such as Whatsapp groups [13], which may be especially important in secondary-level facilities where specialized support is less available.

In both tertiary- and secondary-level health facilities, meeting the basic requirements of infrastructure, nursing support, and clinical oversight is necessary for the safe and effective scale-up of bubble CPAP.

### Strengths and limitations

To the best of our knowledge, this is the first study that directly compares barriers and facilitators to the scale-up of bubble CPAP between tertiary and secondary-level facilities in a LMIC. The strength of this study is exploring implementation factors for bubble CPAP in both tertiary- and secondary-level facilities in sub-Saharan Africa and a focus on understanding barriers and facilitators as the main objective. Though most of the literature on bubble CPAP use in sub-Saharan Africa was conducted in tertiary level facilities, some studies on its implementation in secondary-level facilities are now emerging [24–27]. To the best of our knowledge, this is the first study comparing the implementation factors between rural district hospitals and urban central hospitals for bubble CPAP in sub-Saharan Africa.

A limitation of this study is that it is a secondary analysis of data. The primary objective of this study and the focus of the first analysis were barriers and facilitators to scale-up of bubble CPAP at Malawian hospitals. While we collected participant demographics including respondents’ facility level, we did not include explicit questions on whether facility level impacted implementation, and this may limit our dataset. Additionally, our study explores the perspectives of healthcare workers on their experiences of using bubble CPAP but does not follow individual cases to evaluate the proportion of eligible neonates that actually received bubble CPAP. Consequently, it may seem that the frequency of use is low at the district level, but it is unclear if it is because district staff are not using CPAP, or if there are simply higher numbers of critical cases at the tertiary hospital. Because there may be an initial challenge of initiating babies on bubble CPAP at district level facilities, barriers and facilitators to implementation may shift as district health workers gain confidence in initiation.

## Conclusion

While this study specifically looked at the experience of bubble CPAP use at tertiary- and secondary-level hospitals in Malawi, it highlights the need to tailor implementation to different health level settings for scaling up of innovative health technologies more broadly. Policies that consider health system contexts and the basic need for staff to feel comfortable, empowered, and supported in its use as well as value its effectiveness are important for effective implementation.

## Supplementary Information

**Additional file 1.** Consolidated Criteria for Reporting Qualitative Studies (COREQ) checklist.

**Additional file 2.** Interview topic guide.

## Data Availability

Additional file 1 - Consolidated Criteria for Reporting Qualitative Studies (COREQ) checklist Additional file 2 – Interview topic guide The datasets generated and/or analyzed during the current study are not publicly available due to participant privacy but are available from the corresponding author on reasonable request.

## Abbreviations

CHAM: Christian Health Association of Malawi
CIHR: Canadian Institutes for Health Research
CPAP: Continuous positive airway pressure
DHMT: District health management team
DHO: District health officer
DMO: District medical officer
DNO: District nursing officer
GAC: Global Affairs Canada
HIE: Hypoxic ischemic encephalopathy
IMCHA: Innovating for Maternal and Child Health in Africa initiative
IDRC: Canadian International Development Research Centre
SCT: Social cognitive theory
TAM: Technology acceptance model
