# Supplementary material for: Scaling up newborn care technologies from tertiary- to secondary-level hospitals in Malawi: an implementation case study of health professional perspectives on bubble CPAP
Source: Implement Sci Commun. 2020 Nov 4;1:100. doi: 10.1186/s43058-020-00092-8 (PMC7640662; doi:10.1186/s43058-020-00092-8)
Supplement: Supplementary file 2 — Additional file 2. Interview topic guide. [file 43058_2020_92_MOESM2_ESM.docx]

**Healthcare workers experiences with bubble CPAP in neonatal nurseries**

TOPIC GUIDE

TRAINING ON CPAP

1. First of all, could you tell me more about the time when you first started to use CPAP?

- Did you receive formal training/refresher training or did you learn on the job?
- Did you feel this training/learning on the job was sufficient to prepare you for the work you do?
- If not, were there other things regarding CPAP that you wish to learn?

CPAP AT WORKPLACE

1. I have a couple of general questions about using CPAP for newborn babies at your workplaceHow is CPAP initiated at your unit?
   - Who has the authority to start CPAP?
   - Is this different on day/night shifts, different depending on the staff available?

- Once the decision has been made, who is responsible for applying CPAP?
- Once the CPAP is applied, can you describe who monitors the baby and how this task is done?
  - What kind of monitoring is ideally needed?
  - In your experience, is this always possible? If not, why?

1. Can you describe a time at your workplace when CPAP was indicated for a baby but it was not given/ it was given but with a substantial delay?

- Why do you think this happened?
- If there was a delay – is there anything that you think can be done differently to ensure that neonates who require CPAP receive it promptly?
- Apart from the situation you have just described, do you find there are other circumstances when CPAP is not given although the baby needs it or given with a delay? Tell me more.

1. Have you ever observed any disagreements about whether CPAP should be used or not?

- Can you describe the situation? Who disagreed?
- Why do you think disagreement happened?
- Was the disagreement resolved?
- Apart from the situation you have just described, do you find there are other circumstances when there is disagreement about CPAP? Tell me more.

PERCEPTIONS OF CPAP EFFECTIVENESS

1. In your experience, have you seen situations where CPAP helped the baby get well?

- What happened?
- What went well?

1. In your experience, have you seen situations where you felt CPAP caused harm to the baby?

- What happened?
- What was the reason for harm?
- Did this change your opinion on the use of CPAP and its effectiveness?

PERSONAL EXPERIENCE WITH CPAP

1. Do you experience any challenges with initiating CPAP/working with babies on CPAP (depending on the role of the person interviewed) in your present job?

- Tell us about the specific challenges
- Who can you ask for support?

PERCEPTIONS OF PARENTAL UNDERSTANDING AND PARTICIPATION IN CARE OF BABIES ON CPAP

1. When a newborn is started on CPAP, how do you help the mother/other caretaker understand why the baby is on CPAP?

- Who talks to parents? When?
- If no one talks to parents, why not
- If you are the one talking to them, what would you say? Can you give me an example?
- If parents don’t understand, do you use any other ways to explain what is going on? Can you give me an example?

1. Have you ever had an experience when parents refused CPAP for the baby?

- Who refused? Mother, guardian, father, other
- In your understanding, why did parents refuse?

1. Have you had experience trying to convince parents who initially refused to use CPAP?

- Did they change their mind?
- Why? Why not?

1. In your experience, how do parents feel about holding/changing/feeding the baby on CPAP?

- Does having baby on CPAP stop them from doing some of these activities?
- In your experience, do parents whose baby is on CPAP require a lot of additional support to take care of the baby?
- Is there anything you do to support parents to be able to take care of their baby while on CPAP? Can you give me an example? What would you say/do?

CLOSING

1. Thank you. These are all the questions that I had for you. Is there anything more you would like us to know about your experience with CPAP or how health care workers could be supported to continue to provide CPAP to newborns?
